# Choroid plexus-cerebrospinal fluid route for monocyte-derived macrophages after stroke

**DOI:** 10.1186/s12974-017-0909-3

**Published:** 2017-07-28

**Authors:** Ruimin Ge, Daniel Tornero, Masao Hirota, Emanuela Monni, Cecilia Laterza, Olle Lindvall, Zaal Kokaia

**Affiliations:** Laboratory of Stem Cells and Restorative Neurology, Lund Stem Cell Center, University Hospital, SE-221 84 Lund, Sweden

**Keywords:** Stroke, Inflammation, Choroid plexus, Transplantation, Functional recovery

## Abstract

**Background:**

Choroid plexus (CP) supports the entry of monocyte-derived macrophages (MDMs) to the central nervous system in animal models of traumatic brain injury, spinal cord injury, and Alzheimer’s disease. Whether the CP is involved in the recruitment of MDMs to the injured brain after ischemic stroke is unknown.

**Methods:**

Adult male C57BL/6 mice were subjected to focal cortical ischemia by permanent occlusion of the distal branch of the right middle cerebral artery. Choroid plexus tissues were collected and analyzed for Vcam1, Madcam1, Cx_3_cl1, Ccl2, Nt5e, and Ifnγ expression at different timepoints after stroke using qPCR. Changes of MDMs in CP and cerebrospinal fluid (CSF) at 1 day and 3 days after stroke were analyzed using flow cytometry. Infiltration of MDMs into CP and CSF were validated using β-actin-GFP chimeric mice and Fgd5-CreERT2 x Lox-stop-lox-Tomato mice. CD115+ monocytes were isolated using a magnetic cell separation system from bone marrow of Cx_3_cr1-GFP or wild-type C57BL/6 donor mice. The freshly isolated monocytes or M2-like MDMs primed in vitro with IL4 and IL13 were stereotaxically injected into the lateral ventricle of stroke-affected mice to trace for their migration into ischemic hemisphere or to assess their effect on post-stroke recovery using open field, corridor, and active avoidance behavioral tests.

**Results:**

We found that CP responded to cortical stroke by upregulation of gene expression for several possible mediators of MDM trafficking and, concomitantly, MDMs increased in CP and cerebrospinal fluid (CSF). We then confirmed that MDMs infiltrated from blood into CP and CSF after the insult using β-actin-GFP chimeric mice and Fgd5-CreERT2 x Lox-stop-lox-Tomato mice. When MDMs were directly administered into CSF following stroke, they homed to the ischemic hemisphere. If they had been primed in vitro prior to their administration to become M2-like macrophages, they promoted post-stroke recovery of motor and cognitive function without influencing infarct volume.

**Conclusions:**

Our findings suggest the possibility that autologous transplantation of M2-like MDMs into CSF might be developed into a new strategy for promoting recovery also in patients with stroke.

## Background

Ischemic stroke is caused by occlusion of a cerebral artery, leading to focal ischemia and cell death. Although most survivors improve to some degree, stroke is a leading cause of long-term disability in humans and effective therapies to promote recovery in the chronic phase are lacking [11]. Recent studies in animal models have indicated that monocyte-derived macrophages (MDMs) play an important role for the spontaneous functional restoration. Following a stroke, resident microglia are activated and MDMs and other blood-borne leukocytes are recruited to the ischemic hemisphere [8, 22]. Infiltration of MDMs occurs mainly over the first week and peaks at 3 days after the insult both in a transient [35] and a permanent ischemia model [21] in mice. We have shown that ablation of monocytes during the first week after stroke abolishes long-term behavioral recovery [35].

Macrophages are known to express a wide spectrum of activity, ranging from pro-inflammatory (M1-like) to anti-inflammatory (M2-like) state, depending on their microenvironment [20]. After myocardial infarction, pro-inflammatory M1-like macrophages are the dominant phenotype in the early phase digesting damaged tissue, followed by dominance of anti-inflammatory M2-like macrophages that promote healing in the second phase [23]. Recently, it was reported that myeloid-derived growth factor, produced by monocytes and macrophages, promotes repair after myocardial infarction [15]. Similar to the heart, infiltrating anti-inflammatory M2-like MDMs were shown to improve recovery following injury to the mouse spinal cord [29]. Over the first week following an ischemic stroke in mice, MDMs exhibit a similar switch from pro-inflammatory to anti-inflammatory activity [35]. Interestingly, Hu and colleagues found that expression of M2 markers peaked earlier than that of M1 markers [14]. However, these authors did not distinguish microglia from blood-borne macrophages. Anti-inflammatory M2-like MDM-derived factors, such as Ym1 and TGFβ, were proposed to contribute to the observed spontaneous post-stroke recovery. In line with these animal experimental data, the level of a subtype of monocytes, which expresses surface markers similar to those of mouse anti-inflammatory macrophages [12], was inversely related to poor outcome in stroke patients [34].

Blood-borne leukocytes can enter the diseased central nervous system (CNS) not only via a leaking blood-brain barrier but also through other routes. For example, effector T cells were found to infiltrate the CNS parenchyma via the leptomeninges in a rat multiple sclerosis model [28]. Apart from its canonical function of secreting cerebrospinal fluid (CSF), the choroid plexus (CP) has recently been reported to mediate both immunosurveillance of the healthy CNS and trafficking of leukocytes in several CNS diseases [16, 17, 26, 29]. After traumatic brain injury, CP mediates infiltration of blood-borne monocytes and neutrophils into the lesion site [31, 32]. CP plays a similar role in Alzheimer’s disease, and manipulating this selective gateway for immune cells results in increased infiltration of MDMs and clearance of amyloid plaques [1, 2]. Importantly, it has been shown that CP specifically orchestrates recruitment of monocytes that give rise to beneficial M2-like MDMs in the injured area of the spinal cord, whereas monocytes that enter through the leptomeninges display M1-like activity at the lesion site [29]. Whether CP is involved in the recruitment of MDMs to the injured brain after ischemic stroke is unknown.

Here, we show that CP responds to an ischemic stroke located in the cerebral cortex by upregulation of gene expression for some adhesion molecules and chemokines concomitantly with an increase of monocytes in CSF. We demonstrate that MDMs can infiltrate from blood into CP and CSF after stroke. We then provide evidence that both freshly isolated monocytes and in vitro-polarized M2-like macrophages delivered in CSF can migrate into the ischemic hemisphere and that the polarized M2-like macrophages improve motor and cognitive function without influencing infarct volume. Our findings highlight the importance of M2-like MDMs, which reach CSF and infiltrate the injured parenchyma, for functional restoration after stroke.

## Methods

### Animals

All procedures were carried out in accordance with the guidelines set by the Malmö-Lund Ethical Committee for the use of laboratory animals and were conducted in accordance with the European Union directive on the subject of animal rights. Procedures were carried out on C57BL/6 mice (25–30 g, Charles River, Germany), β-actin-GFP mice (25–30 g, The Jackson Laboratory stock No. 006567), Fgd5-CreERT2 mice (25–30 g, The Jackson Laboratory stock No. 027789), Lox-stop-lox-Tomato mice (25–30 g, The Jackson Laboratory stock No. 007909), and male Cx3cr1-GFP mice (The Jackson Laboratory stock No. 005582), housed under 12 h light/12 h dark cycle with ad libitum access to food and water. For generation of chimeric mice, wild type C57BL/6 recipient mice received 900 cGy irradiation (Cesium source). Bone marrow cells isolated from femur, tibia, and hips of β-actin-GFP mice were then intravenously injected into the tail vein of recipient mice. Transplanted mice were kept under antibiotic treatment in drinking water. Successful generation of chimeric mice was confirmed 2 weeks later by FACS analysis of peripheral blood. Fgd5-CreERT2 x Lox-stop-lox-Tomato mice were generated by crossing Fgd5-CreERT2 mice with Lox-stop-lox-Tomato mice and treated with tamoxifen for over 1 year in order to label all hematopoietic stem cell-derived cells.

Choroid plexus tissue was collected at 6 h, 1 day, 3 days, 7 days, and 14 days after stroke from male C57BL/6 mice and analyzed using qPCR. For delivery of Cx_3_cr1-GFP non-primed freshly isolated monocytes or primed M2-like MDMs into lateral ventricle, injection was carried out 1 day after stroke on male C57BL/6 mice and analysis was performed 3 days after transplantation (4 days after stroke).

### Surgical procedures

Animals were anesthetized with isoflurane (3.0% induction; 1.5% maintenance) mixed with air_._ All animals received locally injected marcaine for pain relief. While under anesthesia and in the early recovery period (2 h), animals were placed on a heating pad maintained at 37 °C.

Permanent occlusion of the distal branch of the right middle cerebral artery (dMCAO) was performed on adult 25–30 g C57BL/6 mice under anesthesia as described elsewhere [25]. In brief, after shaving the skin, a scission was made between the right eye and the right ear. Muscles covering the cranium were cut and opened, and a small hole was then drilled in the cranium at the level of the distal portion of the right middle cerebral artery. The dura mater was removed, and the artery was visualized and occluded by cauterization. The artery was then cut off to make sure there was no remaining blood flow to the corresponding cortical region. After the skin had been sutured, mice were injected with 1.5 ml Ringer’s solution, returned to their cages, and put on a heating pad. For sham-operated mice, the distal portion of the middle cerebral artery was exposed in the same way as in dMCAO surgery, but without occlusion of the artery using cauterization.

For macrophages administration, phosphate-buffered saline (PBS) solutions with or without cells (vehicle) were stereotaxically injected using a glass microneedle into the lateral ventricle (coordinates: −0.1 mm rostral, −1.0/+1.0 mm lateral to bregma, 2.2 mm ventral from brain surface). Injection was carried out 1 day after MCAO. Mice were randomly allocated to Cell or PBS groups using a random sequence generated (https://www.random.org/sequences/). In total, 5 μl PBS with or without 3 million cells were injected using a speed of 1 μl/min. Microneedle was left in place for 5 min after all solution had been injected, and was then slowly removed in 1 min. Finally, wound was cleaned and sutured, and mice were returned to cages with heating pad.

### Choroid plexus tissue and cerebrospinal fluid collection

For choroid plexus (CP) tissue collection, mice were deeply anesthetized with an overdose of pentobarbital and transcardially perfused with at least 150 ml 4 °C saline to thoroughly remove blood from CP. Brain was removed, and CP tissues were collected under surgical microscope. CP in the 4th ventricle was first collected, followed by the ones in 3rd ventricle and in lateral ventricles. CP tissues were then transferred into pre-cooled Eppendorf tube on dry ice for RNA collection or in 4 °C L-15 medium for FACS analysis. For immunohistochemical analysis of CP, freshly isolated tissue was fixed in 4% paraformaldehyde (PFA) overnight and washed in PBS for further analysis.

Cerebrospinal fluid (CSF) was collected from cisterna magna [18]. In brief, mice were anesthetized using isoflurane (3.0% induction; 1.5% maintenance) mixed with air, and were then fixed on a stereotaxic frame. A scission over the back of the neck was made, and muscles covering cisterna magna were separated using a pair of cotton anti-bleeding bars. Body of the mouse was bent at 135° to the head. Cisterna magna was visualized under microscope. A glass pipette was carefully inserted into cisterna magna with avoidance of arteria spinalis dorsalis. On average, 2 μl CSF were collected from each mouse. Any CSF contaminated with blood was discarded. The collected CSF was blown out from the glass pipette using a syringe and was kept at 4 °C for further analysis.

### Immunohistochemistry and microscopical analysis

Mice were deeply anesthetized with an overdose of pentobarbital and transcardially perfused with saline followed by 4% PFA. Brains were post-fixed overnight in 4% PFA and then placed in 20% sucrose for 24 h before coronal sectioning (30 μm) on dry ice. Brain sections were kept in anti-freezing solution before staining. For the staining, free-floating sections were washed in KPBS lasting 10 min for three times and then pre-incubated with the appropriate serum for 1 h. Sections were then incubated with primary antibodies overnight at 4 °C. After incubation, sections were washed in KPBS with 0.25% Triton X-100 lasting 10 min for three times, then incubated for 2 h in the dark with secondary antibodies conjugated with Cy3, Alexa Fluor 647 or Alexa Fluor 488 (1:200, Molecular Probes, Life Technologies). The following antibodies were used: goat anti-CD206 (1:100, R&D), rat anti-VCAM1 (1:50, Santa Cruz), rat anti-CD11b (1:200, AbD Serotec), chicken anti-GFP (1:400, Merck Millipore), rat anti-CD16/32 (1:200, BD Biosciences), rabbit anti-YM1 (1:100, Abcam), rabbit anti-NeuN (1:2000, Abcam), rabbit anti-IGF1 (1:50, Abcam), rabbit anti ZO-1 (1:200, Life Technologies), rabbit anti-IBA1 (1:1000, Wako), Cy3, Alexa Fluor 647 or Alexa Fluor 488-conjugated donkey anti-chicken, goat, rat or rabbit (all 1:200, Jackson ImmunoResearch), and biotinylated horse anti-rabbit (1:200, Vector Laboratories). Nuclei were then stained with Hoechst 33342 (1:4000, Molecular Probes or Jackson Laboratories) for 10 min followed by three rinses and sections were mounted with Dabco (Sigma) on gelatin-coated slides.

Single labeling for NeuN was performed with biotinylated horse anti-rabbit antibody and visualized with avidin-biotin-peroxidase complex (Elite ABC kit, Vector Laboratories), followed by peroxidase-catalyzed diaminobenzidine reaction.

Visualization of IBA1+/CD206+, IBA1+/CD16/32+, GFP+/CD206+, GFP+/CD16/32+, GFP+/YM1+ double positive cells was done using a confocal microscope (ZEISS LSM 780, Zees, Germany). Pictures were taken using ZEN software (Zeiss, GERMANY).

For infarct volume estimation, area of uninjured tissue in ipsilateral and contralateral hemisphere was measured using Visiopharm software (Visopharm, Denmark). The area of uninjured tissue in the ipsilateral hemisphere was subtracted from that of the contralateral hemisphere to get infarct area, and this infarct area was subsequently multiplied with section thickness (30 μm) and series numbers (8 series) to get infarct volume. The assessment was performed in a blinded manner.

### Flow cytometry

Choroid plexus tissue was diced and re-suspended in a +37 °C papain, neutral protease (dispase II), DNAse (PPD) solution and incubated for 30 min at +37 °C. The PPD solution was prepared as follows: 2.5 U/ml papain (Worthington Biochemical Corporation), 250 U/ml DNAse I (Worthington Biochemical Corporation), and 1 U/ml dispase II (Roche) were dissolved in DMEM containing 4.5 g/l glucose at +37 °C, filter sterilized and stored at −20 °C prior to use. Tissue was then triturated, and excess DMEM/F12 with glutamine (500 μl/50 ml) and 10% fetal bovine serum (FBS) medium was added. Cells were washed by centrifugation, re-suspended in FACS block buffer (2% FBS in PBS), and strained through a 40-μm strainer. Cells were re-centrifuged and re-suspended in FACS block buffer with CD16/32 antibody (1:100, BD Biosciences) for 10 min at +4 °C. Cells were then incubated with antibodies for 30 min at +4 °C. Brilliant Violet 421-conjugated rat anti-mouse/human CD11b (1:100, BioLegend) and Brilliant Violet 510-conjugated rat anti-mouse CD45 (1:100, BioLegend) were used. Cells were washed by centrifugation at +4 °C and re-suspended in 200 μl FACS buffer (1% BSA in PBS) to be ready for FACS analysis (BD FACS LSRII, Becton Dickinson, Franklin lakes, NJ). Because of the small volume of CSF samples, 20 μl FACS block buffer were first added to CSF. Then, the samples were incubated with antibodies as mentioned above. After incubation, 100 μl FACS buffer were added. DRAQ5 (1:200, Thermo Scientific) and 2 μl propidium iodide (PI) were added to the CP and CSF samples before analysis for the identification of live cells.

### RNA extraction and quantitative PCR

**Fig. 1:**
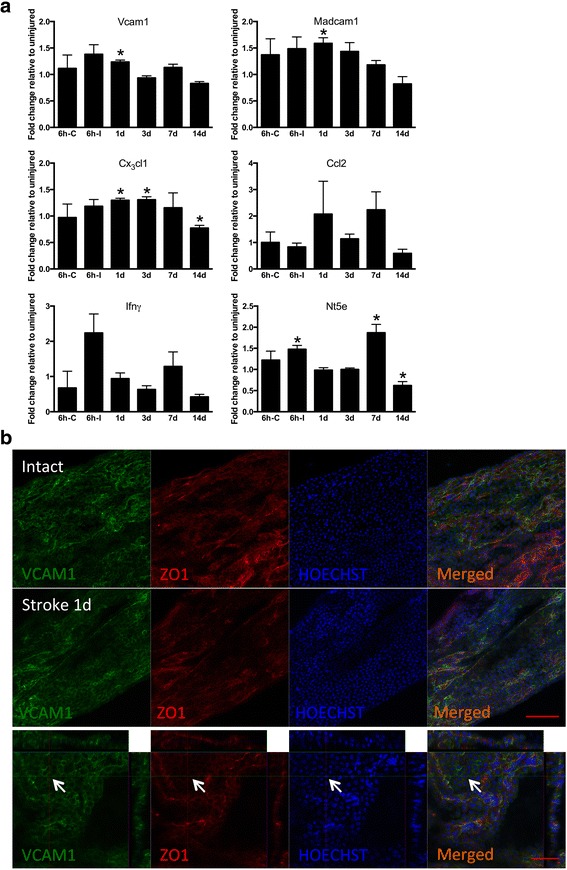
Cortical stroke upregulates adhesion molecule and chemokine gene expression in choroid plexus (CP). **a** qPCR analysis of adhesion molecule and chemokine gene expression in choroid plexus (CP). The sample values from MCAO-subjected mice were normalized to and presented as fold-change relative to the corresponding uninjured mouse sample values in each plate. Measurements at different time-points after the insult using qPCR were performed on pooled CP tissue from both lateral ventricles and third and fourth ventricle except at 6 h when CP ipsi- and contralateral to the ischemic lesion were analyzed separately. Means ± SEM. *Asterisk*: significant difference compared to corresponding samples from uninjured animals, unpaired *t* test, *n* = 4–5. 6 h-C = 6 h contralateral and 6 h-I = 6 h ipsilateral to injury. **b** Expression of VCAM1 protein on ZO-1 positive CP epithelium in intact mice and at 1 day after stroke. Scale bar 100 μm (*upper images*), 50 μm (*lower images*). *Arrows* depict colocalization of VCAM1 with ZO-1

Total RNA was extracted from cells or tissue using a RNeasy Plus micro kit (Qiagen) and then reversed to cDNA using a qScript cDNA Synthesis Kit (QuantaBio). For quantitative PCR, TaqMan Gene expression master mix (Life Technologies) and TaqMan probes (Life Technologies) were used (Table 1). Cycle threshold values of target genes were normalized to geometric mean of housekeeping HPRT and GAPDH to get ΔCt. Two to the power of −ΔCt were calculated for final analysis. Because choroid plexus samples collected from different time-points after MCAO (6 h, 1d, 3d, 7d, and 14d) were run at different plates, MCAO sample values were normalized to the corresponding uninjured mouse sample values in each plate and presented as fold change relative to uninjured (Fig. 1).

**Table 1:** Taqman probes used for qPCR analysis

Gene name	Gene function	Taqman probe number
Hprt	Housekeeping gene	Mm03024075_m1
Gapdh	Housekeeping gene	Mm99999915_g1
Nt5e	Leukocyte transmigration mediator	Mm00501910_m1
Ifnγ	Choroid plexus activator	Mm01168134_m1
Ccl2	Chemokine	Mm00441242_m1
Cx_3_cl1	Chemokine	Mm00436454_m1
Madcam1	Adhesion molecule	Mm00522088_m1
Vcam1	Adhesion molecule	Mm01320970_m1
Tnfα	M1-like macrophage marker	Mm00443258_m1
Igf1	M2-like macrophage marker	Mm00439560_m1
Ym1	M2-like macrophage marker	Mm00657889_mH
Tgfβ1	M2-like macrophage marker	Mm01178820_m1
Arg1	M2-like macrophage marker	Mm00475988_m1

### Monocyte isolation and culture

Bone marrow cells were collected from male Cx_3_cr1-GFP or wildtype C57BL/6 donor mice by crushing the femurs, tibiae, and hips. Cells were passed through a 40-μm strainer and rinsed with PBS supplemented with 2% FBS. CD115+ cells were isolated using a magnetic cell separation system and biotinylated anti-CD115 antibody combined with streptavidin-magnetic beads (Miltenyi Biotec, Germany). The freshly isolated monocytes were used for direct transplantation or further culture.

For M2-like macrophage generation, monocytes were isolated as mentioned above and then cultured at 0.5 × 10^6^ cells/ml in RPMI-1640 medium supplemented with 10% FBS, 1 mM l-glutamine, 1 mM sodium pyruvate, 100 U/ml penicillin, 100 mg/ml streptomycin, 50 ng/ml M-CSF (Peprotech), 50 ng/ml IL4 (Peprotech), and 25 ng/ml IL13 (Peprotech). Five days later, macrophages were collected, washed, and resuspended in PBS for transplantation.

### Behavioral test

All behavioral tests were done in a blinded manner.

For open field test, mice were brought into the test room 30 min before the test to acclimate the mice to the environment. During the whole 1-h test, the test room was kept in darkness. For the test, mice were kept in a black box equipped with a camera. Movements of the mice were automatically recorded using ANY-MAZE software (Stoelting Co., UK). Various parameters of mice movement, such as total movement distance, time spent on movement (Time immobile), mobile episode number, clockwise rotation number, anti-clockwise rotation number, time freezing, rearing number, and central zone entries number, were obtained automatically by the software. Twelve sessions of individual 5-min test were acquired and averaged for further analysis.

The corridor test adapted to mice was used to assess sensorimotor impairment [6, 13]. Briefly, animals were food deprived 12 h before the first testing day and kept on a restricted food intake (2.5–3.5 g/day) so that the body weight did not fall below 85% of initial value. Food was provided only after the daily test session. At the first time point, mice were habituated to the corridor by scattering sugar pellets along the floor and allowing them to freely explore for 10 min on 2 consecutive days before testing. When testing began, the mice were transferred to one end of the testing corridor. The numbers of ipsilateral and contralateral retrievals were counted until a maximum time of 5 min had elapsed. A “retrieval” was defined as an exploration into a pot, whether or not a pellet was eaten, and a new retrieval could only be made by investigating a new pot. Two sessions of the test were done for each mouse in one test day. Retrieval average was calculated for the total five testing days. Contralateral side touches (% of total) were expressed as percentage of pellets eaten or smelled on the contralateral side out of those on contralateral and ipsilateral sides combined.

The cognitive function of mouse was assessed based on their escape behavior [27] in an automated step-through type system (GEMINI, San Diego Instruments Inc., San Diego, CA, USA). The equipment software controls the opening of the gate between the two boxes and the electrical shocks on grid floor. On the first day (pre-training day), the tested mouse was allowed to habituate to the environment with both boxes in darkness and gate opened for 10 min. For active avoidance test training, the light turned on in the box opposite to where the mouse was. When the mouse stayed in the dark box over 30 s, it received an electrical shock of 1 mA for 5 s. This was repeated 30 times during the first training day, and the number of moving to the illuminated box was counted as the number of avoidances. This training was performed in all mice for a total of 3 days in the same way. Five days after last day of training, mice were tested in the same way as in the training but without any electric shock and the number of avoidances was counted. This trial test was repeated for 4 days.

### Statistical analysis

Comparisons were performed using Prism 6 software (GraphPad Software, Inc.) by Student’s unpaired *t* test or two-way ANOVA followed by Bonferroni’s post hoc test. Data are presented as means ± SEM, and differences are considered significant at *p* < 0.05.

## Results

### Cortical stroke upregulates expression of leukocyte trafficking molecules by the brain choroid plexus

In the first experiment, we determined whether brain CP responds to stroke by changing gene expression for factors that might be involved in homing and trafficking of monocytes. We used a model of cortical stroke in mice in which a distal branch of the middle cerebral artery is occluded, resulting in permanent cortical ischemia without reperfusion. This model was chosen because in our standard middle cerebral artery occlusion model [35], we also interrupt blood flow in the anterior choroidal artery, which provides blood supply to the CP in the lateral ventricle. This would result in ischemia in the CP and be a confounding factor in the interpretation of the CP response to a stroke-induced lesion in the brain parenchyma [10].

We used qPCR to measure changes in gene expression at different time-points following cortical stroke. We focused on six genes that were found to be upregulated in CP after spinal cord injury: the adhesion molecules Vcam1 and Madcam1, the chemokines Cx_3_cl1 and Ccl2, Nt5e that mediates macrophage transmigration [29], and Ifnγ that can activate CP for immune surveillance and repair [17, 26]. For the qPCR analysis, we pooled CP from the two lateral ventricles and the third and fourth ventricle. At 6 h after stroke, none of the examined factors showed significant change of gene expression compared to uninjured mice (data not shown). However, when we analyzed CP ipsi- and contralateral to the ischemic lesion separately, we found that Nt5e was upregulated at 6 h on the ipsilateral side (Fig. 1a, Nt5e). At 1 day after stroke, Vcam1, Madcam1, and Cx_3_cl1 showed upregulation (Fig. 1a, Vcam1, Madcam1, and Cx_3_cl1), and this change of Cx_3_cl1 expression remained 2 days later (Fig. 1a, Cx_3_cl1). At 7 days, Nt5e was upregulated compared to uninjured condition (Fig. 1a, Nt5e), but, similar to Cx_3_cl1, showed downregulation at 2 weeks (Fig. 1a, Cx_3_cl1, Nt5e). We detected no changes of gene expression of Ccl2 or Ifnγ at any of the examined time-points (Fig. 1a, Ccl2, Ifnγ). Using immunohistochemistry, we observed VCAM1 protein expression in CP of intact mice and 1 day after stroke (Fig. 1b). Taken together, our findings show that although CP is located distant to the ischemic lesion, it responds to cortical stroke by upregulation of adhesion molecule and chemokine genes, mainly during the first 3 days after the insult.

### Monocyte-derived macrophages infiltrate into choroid plexus and cerebrospinal fluid after cortical stroke

**Fig. 2:**
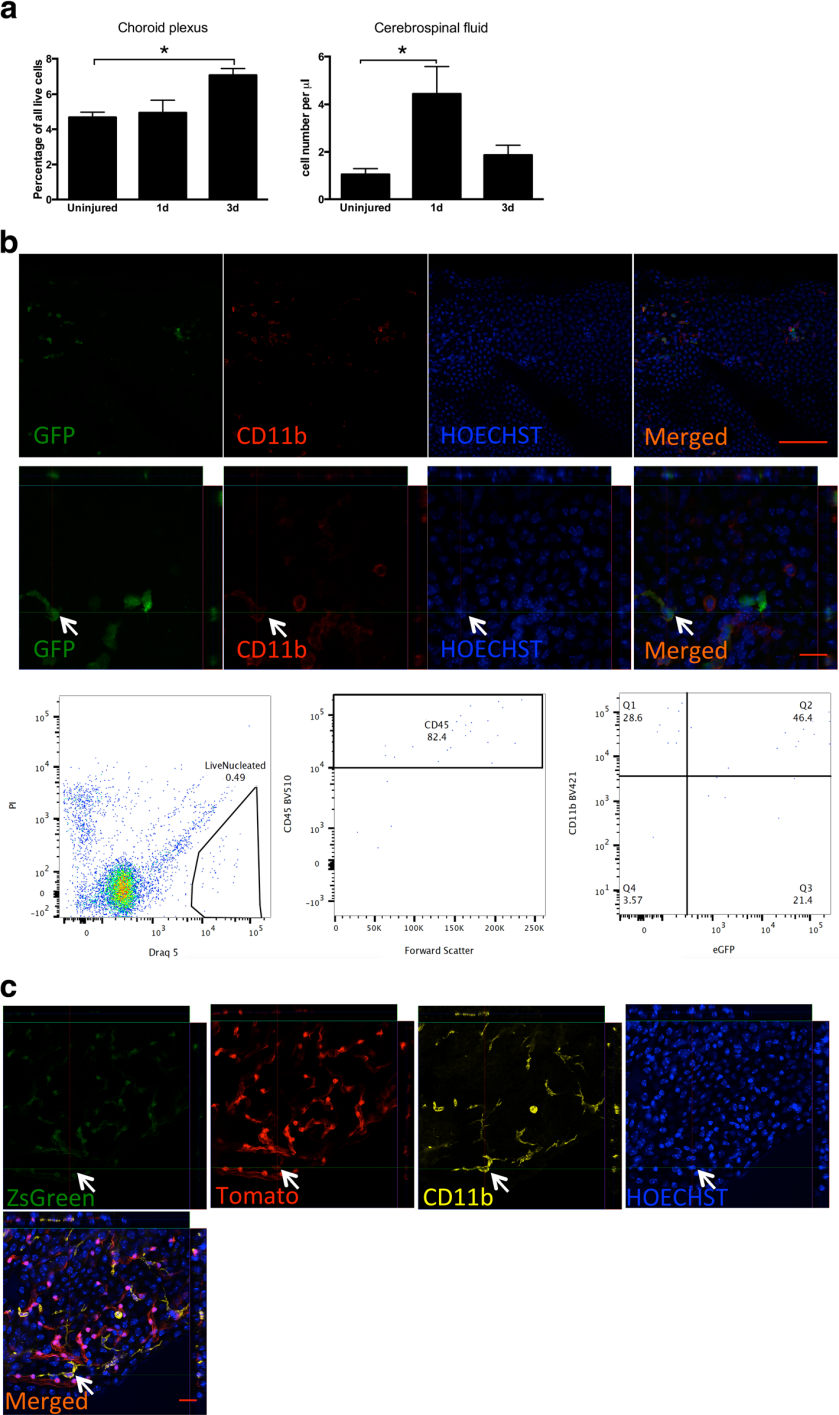
Infiltration of MDMs into choroid plexus (CP) and cerebrospinal fluid (CSF) after cortical stroke. **a** Quantification using FACS of CD45+CD11b+ monocyte-derived macrophages in CP and CSF in uninjured mice, and at 1 day and 3 days after cortical stroke. Means ± SEM. *Asterisk*: significant difference compared to corresponding samples from uninjured animals, unpaired *t* test, *n* = 4–5. **b** Photomicrographs and FACS plots showing infiltration of GFP+CD11b+ MDMs into CP and CSF at 1 day after stroke in β-actin-GFP chimeric mice. Scale bar 100 μm (*upper images*), 20 μm (*lower images*). **c** Photomicrographs showing infiltration of TOMATO+CD11b+ MDMs into CP at 1 day after stroke in Fgd5-CreERT2 x Lox-stop-lox-Tomato mice. Scale bar 20 μm

It has been described that monocyte-derived macrophages (MDMs) are recruited through the CP into the cerebrospinal fluid (CSF) following spinal cord injury [29]. To provide evidence whether such a mechanism can operate also after stroke, we first identified CD45+CD11b+ MDMs in CP and CSF using FACS. We found that the percentage of CD45+CD11b+ MDMs out of the total number of live cells in the CP was increased at 3 days after stroke, whereas at 1 day, it was similar to that in uninjured condition (Fig. 2a). In CSF, the density of CD45+CD11b+ MDMs was increased already at 1 day after stroke, while at 3 days, it had returned to control level (Fig. 2a). These data suggested that MDMs might infiltrate CP and CSF after stroke. To provide further evidence for this hypothesis, we used β-actin-GFP chimeric mice in which blood cells were labeled with GFP. We subjected these chimeric mice to MCAO and, at 1 day after stroke, detected a substantial number of GFP+CD11b+ MDMs in CP and CSF (Fig. 2b). To rule out the possibility that the irradiation procedure per se had induced MDM infiltration into CP and CSF, we utilized Fgd5-CreERT2 mice [7] crossed to Lox-stop-lox-Tomato mice. In this model, Tamoxifen administration leads to Tomato labeling of endothelial and blood cells, which can be distinguished from one another by ZsGreen fluorescence. At 1 day after stroke, we detected, besides FGD5+(ZsGreen+)Tomato+ endothelial cells, also CD11b+Tomato+ZsGreen- MDMs (Fig. 2c). Infiltrating Tomato+CD11b+ MDMs were in close vicinity of Tomato+ vascular endothelium. Taken together, these data provide strong evidence that CP-CSF acts as a possible route for MDM entry into the ischemic brain.

We also analyzed the expression of pro-inflammatory M1-like and anti-inflammatory M2-like markers in macrophages in CP and CSF at 3 days after stroke. Immunohistochemistry showed that almost all Iba1+ macrophages in CP expressed the M1-like marker CD16/32 and 31.9 ± 5.2% the M2-like marker CD206 (Fig. 2b, *n* = 3). Using FACS, we found that 13.3 ± 6.7% (*n* = 3) of all CD45+CD11b+ MDMs were Cx_3_cr1^hi^Ly6C^lo^ M2-like MDMs in CSF. Taken together, these data indicate that MDM could infiltrate into CP and CSF, where some of them express an M2-like marker, following a cortical stroke.

### Monocyte-derived macrophages from CSF home to ischemic hemisphere after cortical stroke

**Fig. 3:**
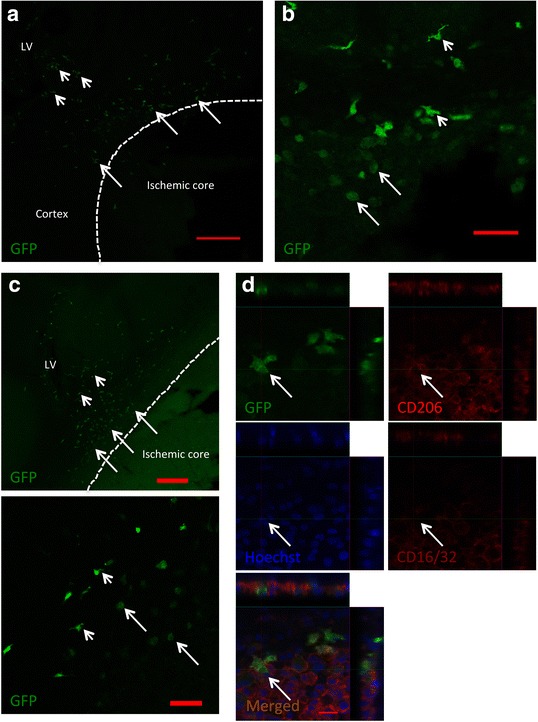
Monocyte-derived macrophages in cerebrospinal fluid (CSF) infiltrate ischemic hemisphere after cortical stroke. **a**–**c** Photomicrographs showing representative images of GFP+ monocyte-derived macrophages (MDMs) in ischemic hemisphere. Monocytes were injected into CSF of ipsilateral (**a**, **b**) or contralateral (**c**) lateral ventricle 1 day after stroke, and the animals were analyzed 3 days later. *Arrows* depict GFP+ MDMs (**a**, **c** *upper image*) and, at high magnification, round MDMs (**b**, **c** *lower image*), in area around ischemic core. *Arrow heads* indicate GFP+ MDMs located between lateral ventricle and ischemic core (**a**, **c** *upper image*) and, at high magnification, ramified MDMs in area around ischemic core (**b**, **c** *lower image*). **d** Photomicrographs showing confocal images illustrating colocalization of GFP with M2-like marker CD206 and M1-like marker CD16/32. *Arrows* indicate GFP+ MDMs (**d**). Scale bar 200 μm (**a**, **c** *upper image*), 50 μm (**b**, **c** *lower image*), 15 μm (**d**). *LV* lateral ventricle

We then asked whether once the monocytes had reached the CSF could they home into the ischemic hemisphere after stroke. To address this, we utilized Cx3cr1-GFP mice in which monocytes are labeled with GFP. We isolated with MACS GFP+ monocytes from the bone marrow using CD115 antibody conjugated with magnetic beads. We directly injected the freshly isolated monocytes into the lateral ventricle ipsilateral to ischemia 1 day after the insult and perfused the mice 3 days thereafter. Using immunohistochemistry, a large number of GFP+ MDMs were found in the area around the ischemic core (Fig. 3a, arrow). Many GFP+ cells, possibly migrating MDMs, were located between the lateral ventricle and the ischemic core (Fig. 3a, arrow head). Some of the GFP+ MDMs in the area around the ischemic core had downregulated GFP and adopted a more round morphology, indicating an activated phenotype (Fig. 3b, arrow), whereas others expressed high GFP protein level and maintained a ramified phenotype (Fig. 3b, arrow head).

We did not find any GFP+ cells in the contralateral hemisphere of stroke-injured animals or, with the exception of a few GFP+ cells around the needle track, in the brains of mice subjected to sham procedure and injected with GFP+ monocytes (data not shown). To further confirm the homing of MDMs, administered into CSF, to the ischemic hemisphere, we injected freshly isolated GFP+ monocytes also into the contralateral lateral ventricle 1 day after stroke. Again we found many GFP+ MDMs in the ischemic hemisphere 3 days later (Fig. 3c). We also injected GFP+ monocytes deficient in Cx_3_cr1 into CSF of ipsilateral lateral ventricle 1 day after stroke and could still detect GFP+ MDMs in the ischemic hemisphere 3 days thereafter. This finding suggests that the Cx3cr1 pathway plays at most a minor role in mediating the migration of MDMs from CSF into the ischemic hemisphere.

We examined the GFP+ MDMs in the area around the ischemic core with respect to their phenotype. Neither the GFP^high^ ramified nor the GFP^low^ round MDMs expressed marker characteristics of M1-like or M2-like phenotype such as CD16/32 or CD206 (Fig. 3d). Taken together, our data using delivery of monocytes into CSF provide evidence that also MDMs which have increased in CSF after cortical stroke can migrate into the ischemic hemisphere after stroke.

### Anti-inflammatory M2-like monocyte-derived macrophages derived in vitro can infiltrate ischemic hemisphere from CSF

**Fig. 4:**
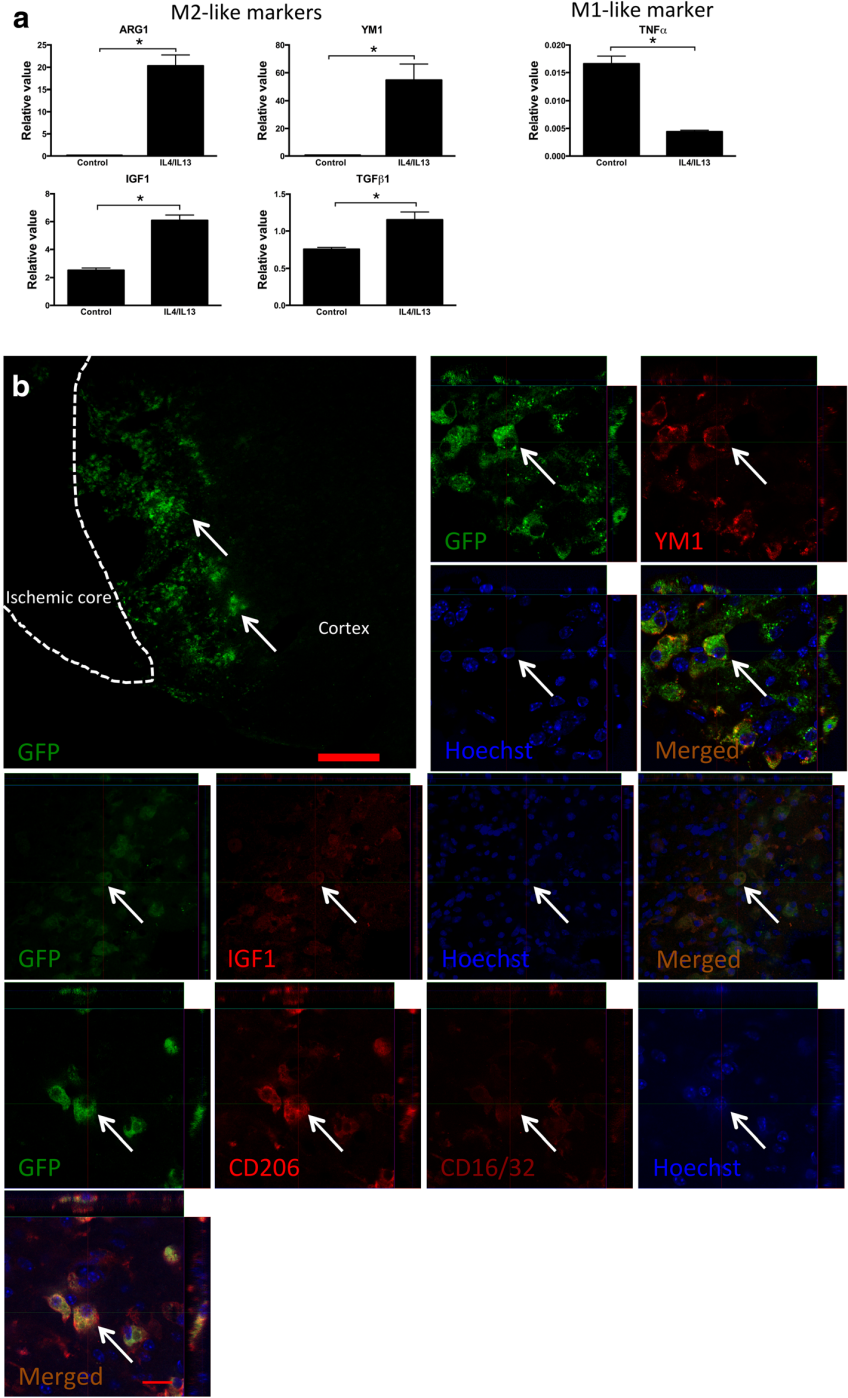
Anti-inflammatory M2-like monocyte-derived macrophages primed in vitro infiltrate ischemic hemisphere from cerebrospinal fluid (CSF). **a** Upregulation of M2-like markers and downregulation of M1-like marker in macrophages cultured with IL4 and IL13 for 5 days as measured using qPCR. Means ± SEM. *Asterisk*: significant difference compared to control macrophages cultured without IL4 and IL13, unpaired *t* test, *n* = 4. **b** Photomicrographs showing infiltration of GFP+ monocyte-derived macrophages co-expressing M2-like markers YM1, IGF1, and CD206 in the ischemic hemisphere at 3 days after injection into ipsilateral lateral ventricle. *Arrows* indicate GFP+ MDMs (**b**). Scale bar 200 μm (**b** *upper left image*), 15 μm (**b** Other)

Next, we investigated if we could take advantage of the CSF route to enhance the infiltration of MDMs that are biased towards anti-inflammatory phenotype prior to their administration to the CSF. Freshly isolated Cx_3_cr1-GFP monocytes from the bone marrow were cultured in the presence of IL4 and IL13 to prime them towards an anti-inflammatory fate. After 5 days in culture, many M2-like markers, such as YM1, ARG1, IGF1, and TGFβ1, were upregulated, whereas the M1-like marker TNFα was downregulated (Fig. 4a). Thus, the freshly isolated monocytes had been activated and adopted an M2-like phenotype. These M2-like MDMs were then injected into the lateral ventricle on the ischemic side at 1 day after cortical stroke, i.e., concomitantly with the observed increase of endogenous MDMs in the CSF at the same time-point (Fig. 2a). Three days later, numerous GFP+ MDMs were found around the ischemic core (Fig. 4b), 47.9 ± 8.5% (*n* = 2) of all ED+ cells are GFP+ M2-like MDMs. Almost all GFP+ MDMs expressed the M2-like markers YM1, IGF1, and CD206, while the M1-like marker CD16/32 was only very weakly expressed, providing evidence that the injected primed MDMs homed to lesion site and maintained their M2-like phenotype at this time-point (Fig. 4b). Taken together, our findings show that M2-like MDMs migrate into the ischemic hemisphere and retain their M2-like phenotype 3 days after delivery into CSF.

### Administration of M2-like monocyte-derived macrophages promotes behavioral recovery after cortical stroke

Finally, we explored if enhancement of the infiltration of M2-like MDMs into the ischemic hemisphere, caused by delivery of the cells into the CSF through the ipsilateral lateral ventricle, could influence the magnitude of behavioral recovery following stroke. Mice were subjected to distal MCAO and randomly allocated to injection of either in vitro-derived M2-like MDMs or vehicle 1 day later. In some animals, we assessed the infarct volume at 7 days after cell or vehicle injection. As expected from our previous work of monocyte depletion after stroke [35], the infarct volume was not affected by the injected MDMs (vehicle: 12.4 ± 2.1 mm^3^, *n* = 5; MDMs: 13.7 ± 1.8 mm^3^, *n* = 6; unpaired t test), indicating that the infiltrating M2-like MDMs had not influenced the extent of ischemic damage.

**Fig. 5:**
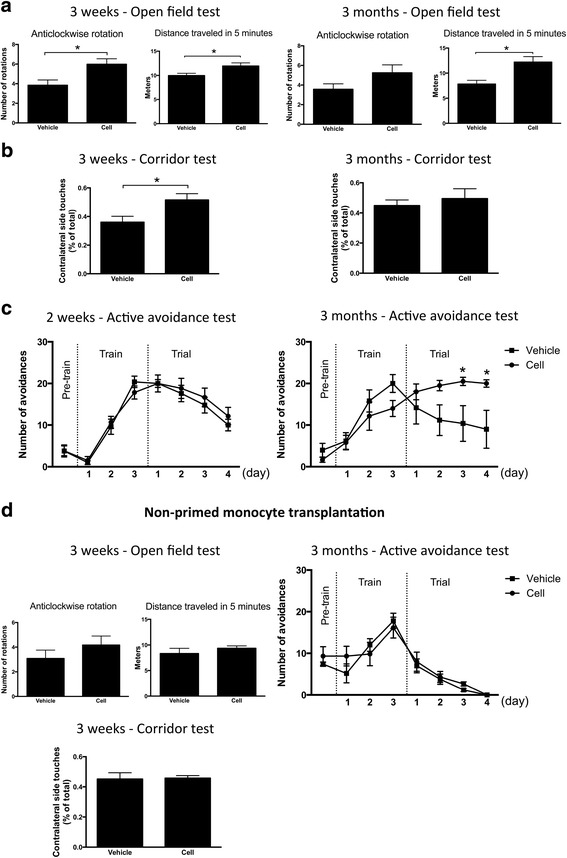
Effects of intraventricular administration of M2-like monocyte-derived macrophages or non-primed monocytes on post-stroke behavioral recovery. Performance in open-field test, corridor test, and active avoidance test at 2 or 3 weeks and 3 months after injection of M2-like monocyte-derived macrophages (*n* = 6, **a**–**c**), or non-primed monocytes (*n* = 6, **d**) or vehicle (*n* = 5, **a**–**d**) into the ipsilateral lateral ventricle 1 day after cortical stroke. Means ± SEM. *Asterisk*: significant difference compared to vehicle-injected animals, unpaired *t* test (**a**, **b** open-field test and corridor test in **d**) and two-way ANOVA with Bonferroni’s post hoc test (**c** active avoidance test in **d**). Contralateral side touches (% of total) indicate percentage of pellets eaten or smelled on the contralateral side out of those on contralateral and ipsilateral sides combined (**b**, corridor test in **d**). Pre-train, Train, and Trial indicate pre-training stage (for 1 day), training stage (for 3 days), and trial test (for 4 days) of active avoidance test, respectively (**c** active avoidance test in **d**)

We then investigated the effect of M2-like MDM delivery into CSF on post-stroke recovery using various behavioral tests: open-field and corridor tests and active avoidance test to assess restoration of motor and cognitive function, respectively. At 3 weeks, mice transplanted with M2-like MDMs showed more pronounced anticlockwise rotation in the open-field test compared to vehicle-injected ones (Fig. 5a), indicating improvement of the deficit in rotational movements previously reported after stroke [5]. Also, cell-injected mice showed increased preference of smelling or eating pellet on the contralateral side in corridor test (Fig. 5b). Notably, at 3 months after transplantation, the difference between the two groups had disappeared (Fig. 5a, b). At both 3 weeks and 3 months, mice that had received M2-like MDMs showed enhanced locomotor activity in the open field test, as evidenced by the increased distance moved (Fig. 5a). In the active avoidance test, there was no difference between cell- and vehicle-injected mice in learning curve at 2 weeks after injection (Fig. 5c). However, at 3 months after injection, mice with M2-like MDMs showed significantly better learning curve than mice with vehicle injection, indicating better memory skills (Fig. 5c). After we finished the behavioral tests at 3 months, we examined the infarct volume between the cell transplantation and vehicle injection groups. Consistent with the result at 7 days after transplantation, the two groups showed no difference in infarct volume (vehicle: 4.84 ± 1.34 mm^3^, *n* = 5; MDMs: 5.14 ± 1.02 mm^3^, *n* = 6; unpaired *t* test).

Next, we then asked whether the in vitro priming of the monocytes to M2-like MDMs before delivery was important for the effect on post-stroke behavioral performance. To this end, we injected either freshly isolated non-primed monocytes or vehicle into the ipsilateral ventricle at 1 day after distal MCAO as in the previous experiment. In contrast to the findings with primed M2-like MDMs, injection of non-primed monocytes did not give rise to any improvement as compared to vehicle in performance of open-field test or corridor test at 3 weeks or of active avoidance test at 3 months after stroke (Fig. 5d). Taken together, these data indicate that intraventricular injection of M2-like MDMs increases post-stroke recovery of both motor and cognitive impairments.

## Discussion

In this study, we have shown that CP responds to a cortical ischemic lesion by transiently elevated gene expression for adhesion molecules and chemokines. Vcam1, Madcam1, Cx_3_cl1, and Nt5e were transiently upregulated up to 1 week after the insult. During the same time period, MDMs increased in CP and CSF, and MDMs could infiltrate from blood into CP and CSF. We then demonstrate that MDMs when delivered into CSF infiltrate the ischemic hemisphere and, if they have been primed in vitro to beneficial M2-like phenotype, promote post-stroke recovery of motor and cognitive function.

Several of the upregulated factors have previously been shown to be essential for MDM infiltration via the CP-CSF route after spinal cord injury [29]. In line with a similar role after cortical stroke, we detected Vcam1, Madcam1, and Cx_3_cl1 upregulation in CP concomitant with increased MDMs in CSF at 1 day following the insult and Cx_3_cl1 was upregulated in CP also at 3 days. The MDMs in the CSF could reflect those that had trafficked through the CP via the transiently upregulated Vcam1, Madcam1, and Cx_3_cl1. The increased density of MDMs in CSF returned to baseline at 3 days after stroke, indicating that the peak of MDM infiltration via CSF route occurs around 1 day following the insult. This time-point is earlier than the peak of overall MDM infiltration into ischemic hemisphere at 4 days after stroke [21]. The upregulation of Vcam1, Madcam1, Cx_3_cl1, and Nt5e in CP following spinal cord injury was maintained at 14 days [29]. In contrast, the response of the CP to cortical ischemia, which was characterized by upregulation of the same factors, was transient and occurred mainly during the first 3 days after the insult. Moreover, we found that only 30% of the total number of Iba1+ macrophages in the CP expressed the M2-like marker CD206 at 3 days after stroke, whereas almost all of them expressed the M1-like marker CD16/32. And 13.3 ± 6.7% (*n* = 3) of all CD45+CD11b+ MDMs were Cx_3_cr1^hi^Ly6C^lo^ M2-like MDMs in CSF at 3 days after stroke. After spinal cord injury, the majority of macrophages in CP were found to be in M2-like state [29]. Taken together, these findings indicate that compared to the role of CP as the main port of entry for M2-like macrophages after spinal cord injury, the CP probably plays only a minor role as a route for M2-like macrophage infiltration after stroke.

We further found that both non-primed monocytes and M2-like macrophages derived in vitro homed into the injured hemisphere after injection into CSF. This finding raises the possibility that once monocytes had reached the CSF, they could home to the lesion site. We did not observe any migration of MDMs into the hemispheres when monocytes were injected into animals which had not been subjected to stroke but to sham procedure, apart from some MDMs left in the needle track. One potential candidate molecule for recruiting MDMs from CSF towards the ischemic lesion is the chemokine CCL2 [33], which was found to be increased in the ischemic cortex of mice and in the CSF of stroke patients [19, 21]. Another potential mechanism is signaling through the Cx_3_cl1 receptor Cx_3_cr1, which is a known chemotactic factor to monocytes/macrophages [9]. Arguing against this possibility, we found that GFP+ monocytes with deficiency in Cx_3_cr1 which were injected into CSF could still migrate into the ischemic hemisphere.

Several lines of evidence have suggested that M2-like macrophages may have a beneficial effect in stroke. For example, M2-like microglia/macrophages were found to protect neurons from ischemic death in vitro [14], and IL-4 promoted long-term recovery after stroke in mice, possibly by inducing an M2-like macrophage phenotype [18]. In contrast, intravenous administration of M2-like macrophages had no effect on motor recovery up to 2 weeks after stroke in rats [4]. However, whether the intravenous injection had resulted in significant infiltration of M2-like macrophages into the ischemic hemisphere was not examined.

We provide here direct evidence supporting a recovery-promoting effect of M2-like MDMs following focal cerebral ischemia. Thus, we show that M2-like MDMs delivered into CSF infiltrate the ischemic hemisphere and promote recovery of both motor and cognitive function after a cortical ischemic lesion. At 3 weeks after the insult, the MDM-injected mice showed more pronounced anticlockwise rotation in the open-field test and higher preference for smelling and eating pellets on the contralateral side in the corridor test as compared to vehicle-injected animals. This difference was transient which may indicate that the MDMs had induced a more rapid recovery of these functional deficits. Locomotor activity was better in cell-injected mice at both time-points, providing evidence of both a rapid and stable improvement. In line with these findings, intrathecal injection of macrophages with M2-like phenotype derived in vitro has been reported to improve neurological performance in stroke patients [3].

We found that although non-primed monocytes could migrate into the ischemic hemisphere, they did not express M2-like markers and had no effect on recovery of motor function at 3 weeks or cognitive function at 3 months post-insult. These data indicate that the M2-like state of MDMs is essential for the recovery-promoting effect observed in our study. This is in agreement with our recent findings following depletion of infiltrating monocytes after stroke [35]. We obtained no evidence that the behavioral improvement after delivery of M2-like MDMs was due to an effect on the extent of the ischemic damage. Therefore, the recovery-promoting effect is most likely due to secreted factors from M2-like MDMs that can promote cellular and synaptic plasticity of remaining neurons. Supporting this hypothesis, we found that at 3 days after transplantation, the M2-like MDMs expressed IGF1, a factor shown to enhance axonal growth of corticospinal motor neurons [24]. Interestingly, we found that at 18 days after delivery of M2-like MDMs into ipsilateral lateral ventricle, hardly any GFP+ M2-like MDMs were detected in the ischemic hemisphere (data not shown). This observation indicates that persistent presence of M2-like MDMs is not necessary for long-term recovery, which is in line with our previous findings showing that depletion of circulating monocytes during the first week after stroke causes impaired functional recovery at 2 months following the insult [35]. Although our data indicate that M2-like MDMs can use the CSF route to infiltrate the ischemic hemisphere and promote recovery, it cannot be excluded that M1-like MDMs, possibly taking other routes such as a leaking blood-brain barrier, convert in situ to M2-like MDMs and contribute to functional restoration. Also, it would be interesting to determine in the future whether CP mediates infiltration also of other leukocytes such as neutrophils and T cells [30, 32] into the stroke-injured brain.

## Conclusions

Our findings suggest the possibility that delivery of M2-like MDMs into CSF could be developed into a new therapeutic strategy for promoting post-stroke recovery. A potential advantage is that M2-like MDMs can easily be obtained from the stroke patient’s own peripheral blood in large numbers, and autologous transplantation into CSF will not require immunosuppressive treatment. Here, we administered 3 million MDMs at 1 day after the insult. However, it will be important in future studies to determine if these cells can give rise to improvement also when delivered at later stages of recovery and to identify the optimum dose of cells for inducing maximum functional restoration. In the clinical translation, autologous human MDMs should be labeled and it should be clarified whether they can efficiently infiltrate the ischemically injured tissue in the stroke patient’s brain and give rise to a measurable therapeutic effect.

## Abbreviations

CP: Choroid plexus
CSF: Cerebrospinal fluid
dMCAO: Distal middle cerebral artery occlusion
GFP: Green fluorescent protein
IL13: Interleukin 13
IL4: Interleukin 4
MDMs: Monocyte-derived macrophages
PBS: Phosphate-buffered saline
PFA: Paraformaldehyde
